# Optimal Sensor Placement of the Artificial Lateral Line for Flow Parametric Identification

**DOI:** 10.3390/s21123980

**Published:** 2021-06-09

**Authors:** Dong Xu, Yuanlin Zhang, Jian Tian, Hongjie Fan, Yifan Xie, Wei Dai

**Affiliations:** 1 School of Automation Science and Electrical Engineering, Beihang University, No.37 Xueyuan Road, Beijing 100191, China; xd@buaa.edu.cn (D.X.); zyl@buaa.edu.cn (Y.Z.); tj960318@buaa.edu.cn (J.T.); xyfan1234@163.com (Y.X.); 2 School of Mechanical Engineering and Automation, Beihang University, No.37 Xueyuan Road, Beijing 100191, China; fanhj@buaa.edu.cn; 3 School of Reliability and System Engineering, Beihang University, No.37 Xueyuan Road, Beijing 100191, China

**Keywords:** underwater robotic fish, artificial lateral line system, sensor placement, feature importance, information redundancy

## Abstract

The multi-sensor artificial lateral line system (ALLS) can identify the flow-field’s parameters to realize the closed-loop control of the underwater robotic fish. An inappropriate sensor placement of ALLS may result in inaccurate flow-field parametric identification. Therefore, this paper proposes a method to optimize the sensor placement configuration of the ALLS, which mainly included three algorithms, the feature importance algorithm based on mean and variance (MVF), the feature importance algorithm based on distance evaluation (DF), and the information redundancy (IR) algorithm. The optimal sensor placement performance selected by this method is verified by simulation. In addition, further experimental verification was conducted using the ALLS. Compared with the uniform sensor placement configuration mentioned in recent studies, the experimental results suggest that the optimal sensor placement method can achieve a more effective prediction of the flow-field parameters, therefore strengthening the underwater robotic fish’s perception and control function.

## 1. Introduction

The lateral line is a collection of sense organs involved in various fish behaviors, such as prey detection, predator avoidance, schooling, intraspecific communication, rheotaxis, station holding, etc. [1,2,3]. The biological lateral line’s sensing unit is the neuromast [4], including superficial neuromasts (SNs) and canal neuromasts (CNs). The SNs stick out of the fish skin [5] and respond to flow velocity [6]. However, CNs are distributed on both sides of the fish body at the same horizontal level [7] and are sensitive to the orientation [6] and water acceleration [7]. Ref [8] suggested a physical explanation for sensory layout across diverse species, which is helpful for the development of follow-up research. Moreover, for environmental studies on fish, [9] has also proposed a robust velocity estimation methodology relevant for studies of fish behavior using a bioinspired fish-shaped artificial lateral line probe (LLP).

To mimic fish’s lateral line canal system, pressure sensors are widely used in recent studies [10,11] to build the artificial lateral line system (ALLS) due to its mature technology and low cost. The multi-sensor ALLS provides sensing and control functions to underwater robotic fish, which can supplement current underwater sensing methods, including sonar and vision [12]. With the feedback from the ALLS, underwater robotic fish can control its direction, flow posture for exploration, interaction, and communication more effectively [13,14].

Currently, ALLS has been used for flow-field information extraction [10] and localization of a vibrating sphere [15]. However, most of the aforementioned studies have adopted the ALLS design with fixed sensor placement according to engineering intuition. The effect of sensor placement on the results is not considered. Moreover, the pressure sensors are evenly distributed on the robotic fish: some are only distributed on the front head of the robotic fish [13], some are evenly distributed on the side of the robotic fish [16], and some are evenly distributed on the side and bottom of the robotic fish [17]. Nevertheless, there is no analytical evidence that these are the best placement. The unreasonable determination of sensor placement based on engineering intuition is the main reason for inaccurate flow-field information monitoring. Therefore, it is necessary to optimize sensor placement for ALLS.

After optimizing the placement of the sensors, it is possible to avoid the loss of information caused by randomly placing pressure sensors in ALLS, resulting in the inaccurate prediction of working conditions [13]. It can also reduce the number of sensors for constructing the ALLS, therefore reducing the cost and power consumption of the underwater robotic fish. Simultaneously, it is avoided that the information extracted by the sensors increases the amount of calculation or detects more redundant information, therefore improving the accuracy of perception [10]. However, only a few pieces of research concentrated on the optimal sensor placement method for the ALLS. Through simulation, [18] optimized the sensor structure and established the pressure distribution models of the lateral surface in uniform flow and turbulent flow. DeVries optimizes sensor placement along a streamlined body using measures of empirical observability [19]. However, this study is only effective in the uniform and steady flow-field. The optimal results are only used for controlling the desired angle of attack with respect to the flow. In Ali Ahraria’s study [15,20,21], a specialized bi-level optimization methodology is proposed to find an optimum design considering the shape and size of the streamlined body and the sensors’ number and locations. However, the above two methods are only applicable to the ALLS composed of hair-sensor arrays. In Siddhartha Verma’s work [22], by combining simulations of the Navier–Stokes equations with Bayesian experimental design, they have determined the optimal arrangements of shear stress and pressure gradient sensors. For pressure sensors, in our previous research [23], we proposed an optimal weight analysis algorithm and a comprehensive evaluation system to optimize the sensor placement for the ALLS.

In this paper, we propose a novel sensor placement optimal method for the ALLS. The optimal placement means that a design maximizes average characterization accuracy when velocity and deflection angle in Uniform flow (UF) and Karman vortex street (KVS) is considered. The contributions of this paper are multifaceted, detailed as follows: (1) a fluid dynamics simulation experiment was carried out through Fluent. In the simulation environment, multiple kinds of investigated scenarios are set up, and a large amount of pressure data are collected, which are used for the research of the optimal placement of the sensors and the information-processing algorithm; (2) an optimal sensor placement algorithm is proposed based on feature importance and information redundancy. Considering these two factors comprehensively, the final optimal sensor position is obtained; (3) the optimized sensor placement is verified using the method of curve fitting of flow-field parameters, i.e., velocity and deflection angle; (4) a comparison with several commonly used sensor placements in the current research has been carried out to verify the effectiveness of the optimal sensor placement algorithm.

This paper is organized as follows. Section 2 introduces the ALLS of the underwater robotic fish and the investigated scenarios set in this paper. Moreover, the experiment platform is presented in detail. The optimal sensor placement algorithm and the evaluation method of different sensor placements are introduced in Section 3. Section 4 shows the result and evaluation of optimal sensor placement. Apart from this, experiments are conducted to verify the effectiveness of this optimal sensor placement algorithm. Section 5 summarizes this paper and provides prospects.

## 2. Description of the ALLS and Experiment

### 2.1. Underwater Robotic Fish with the ALLS

**Figure 1:**
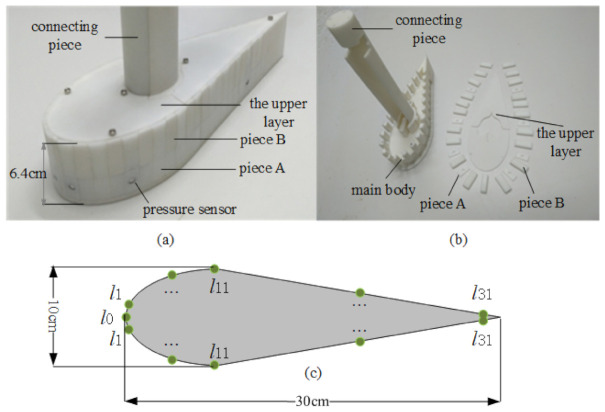
(**a**) Overall appearance of robotic fish. (**b**) Component of the robotic fish. (**c**) Top view of the distribution of pressure sensors on the robotic fish.

Inspired by the robotic fish model [24], the underwater robotic fish is designed according to the adult mackerel’s regular length and size. The robotic fish made by 3D printing is shown in Figure 1. Its shape is a symmetrical cylinder with a fish-shaped cross-section, and the size is about 30cm×10cm×6.4cm. The component of the robotic fish is shown in Figure 1b. The main body, the basic structure of the robotic fish, is made of waterproof nylon material. The main body provides stable support for the robotic fish. The middle layer is designed as a dismountable form so that the placement of the sensor can be easily changed. Two types of resin plates make up the middle layer, piece A and piece B, and piece A is divided into two forms: with holes and without holes. The two types of pieces A have the same size, and the width is about 1cm. Piece B is mainly used for immobilizing piece A. The robotic fish’s upper layer comprises two thin slices, which are fixed on the robotic fish by screws. The function of the connecting piece in Figure 1b is to connect the robotic fish to the mobile rail in the experimental platform. In this paper, the sensors (MS5803-01BA) that construct ALLS can measure pressure data from 100Pa to 0.13MPa with a resolution of 1.2Pa. The sensors are embedded in Piece A with holes, and different sensor placement configurations can be obtained by changing the position of Piece A with holes. In routine underwater measurements, waterproof glue and stainless steel induction caps are used as protective measures.

Considering that the CNs are mainly distributed on the maximum cross-section of biological fish. Therefore, the discussion in this paper is limited to the 2D setting to imitate the fish’s CNs; that is, the pressure sensors are arranged on the same horizontal plane on both sides of the robotic fish, as is shown in Figure 1c. Moreover, the CNs located on the nose of biological fish are quite sensitive to flow-field parameter changes [11]. Therefore, a sensor is placed on the nose of the robotic fish. The remaining sensors are divided into two groups and arranged symmetrically on robotic fish to imitate the biological fish’s symmetry. As the circumference of the robotic fish is about 64cm, and Piece A’s width is about 1cm. Therefore, there are 31 optional sensor positions, symmetrically distributed on both sides of the robotic fish. Due to the limitation of the cost and the robotic fish’s power consumption, the number of sensors in the ALLS is limited. We need to choose the optimal sensors placement position from 31 optional sensors placement positions to construct the ALLS.

### 2.2. Investigated Scenarios

**Figure 2:**
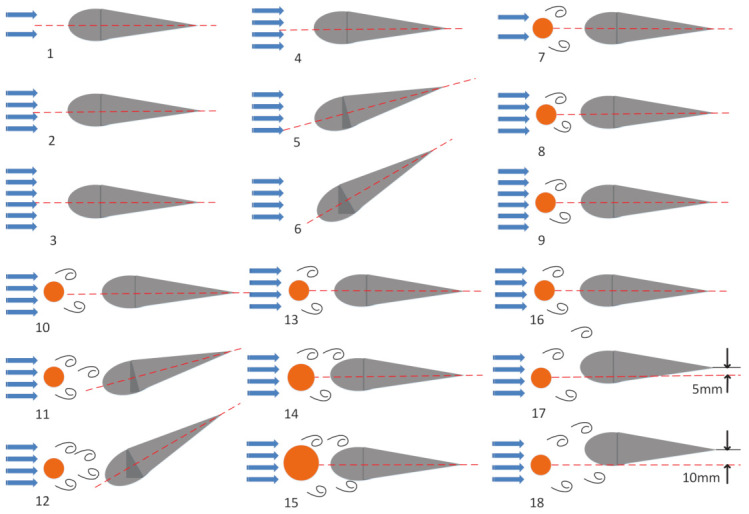
Investigated scenarios.

An underwater robotic fish can change its movement posture when it senses environment change. In light of the investigated scenarios in paper [10], we analyze 18 kinds of scenarios as shown in Figure 2 to simulate the environment in nature, six in the UF, and 12 in the KVS.

First, simulation experiments are conducted in the UF. In the UF, flow speed and deflection angle are two critical parameters. The deflection angle is the angle between the center axis of the robotic fish body and the water flow. As is shown in Figure 2, in the first set of experiments (Figure 2(1–3)), the deflection angle is 0${}^{\circ }$, but the flow speed is different. In the second set of experiments (Figure 2(4–6)), the flow speed is constant, but the deflection angle alters. This paper puts a cylinder in front of the robotic fish to create KVS. By changing the flow speed, the deflection angle, the distance deviates from the center, and the cylinder’s diameter can simulate the different working conditions. For example, in the third set of experiments (Figure 2(7–9)), the robotic fish is placed in the same direction, but the flow speed changes. Then we repeat the simulations for different deflection angles (Figure 2(10–12)) with other parameters fixed. Furthermore, we change the diameter of the cylinder to achieve different working conditions (Figure 2(13–15)). In the end, the simulations about different distances deviated from the vortex center (Figure 2(16–18)) are also added.

As described in Section 2.1, there are 31 optional positions for the pressure sensors. In this paper, the fluid simulation software Fluent is used for simulation experiments, and the pressure data at 31 positions are collected. As the working range of the natural hydrodynamic receptor of fishes is generally 1–150Hz [10]. Therefore, in the simulation experiments, the time step is set to 0.01s (sampling rate of 100Hz). In each experiment, the pressure signal is collected every 0.01s in an observation interval of 4s. Table 1 summarizes the settings and fluid dynamics parameters of the simulation experiment.

### 2.3. Experiment Platform

**Figure 3:**
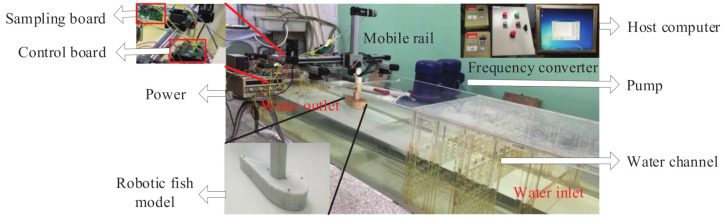
The experiment platform.

As shown in Figure 3, an experimental platform is designed to verify the optimal sensor placement algorithm. The water tank body is made of acrylic resin, and its size is 300cm×60cm×60cm (length × width × height). Two 2Kw pumps power the water tank, and the water flow velocity is adjusted within the range of 0∼30 cm/s through the frequency converter. Although the robot fish’s deflection angle is controlled by the steering gear (DS3115MG). The adjustable range of deflection angle is from $-40^{\circ }$ to $+40^{\circ }$ with the precision is $1^{\circ }$. Several honeycomb boards have been installed perpendicular to the direction of water flow near the water inlet, which helps to form the UF. By placing a cylinder between the robotic fish and the honeycomb boards to generate KVS. The system uses a sampling board based on MCU STM32F103 to samples the pressure data, and a separate control board is used to control the steering gear and the motor.

### 2.4. Experimental Condition

To verify the optimized sensor placement’s validity, we collect experiment data in four pairs of experimental conditions by changing the flow parameters in the flow-field to imitate the flow environment in nature. In each pair of experiments, only one flow parameter, velocity or deflection angle, is changed. The ALLS can predict the changed flow parameter through the fitting models in Section 3. These four pairs of experimental conditions are shown in Table 2 and described as follows:

(1) In the UF, the robotic fish’s axis is coincidental with the water flow, the velocity of the flow-field changes from 1cm/s to 40cm/s.
(2) In the uniform flow-field, the velocity of the flow-field is always 100cm/s, the deflection angle of the robotic fish changes from $0^{\circ }$ to $40^{\circ }$.
(3) Under the condition of the KVS, the cylinder’s diameter is 10cm, the robotic fish’s axis is coincidental with the water flow, the deflection angle of the robotic fish is $0^{\circ }$, the flow velocity changes from 1cm/s to 40cm/s.
(4) Under the condition of the KVS, the cylinder’s diameter is 10cm, the flow velocity is always 100cm/s, the deflection angle of the robotic fish changes from $0^{\circ }$ to $40^{\circ }$.

## 3. Optimization Method for Sensor Placement of the ALLS

The optimization method for sensor placement of the ALLS contains two parts: (1) sensor placement optimal algorithm and (2) Evaluation algorithm to choose the best sensor placement. Through the optimal sensor placement algorithm, we can achieve multiple sensor placement design by changing three weight factors that correspond to the feature importance algorithm based on mean and variance (MVF), the feature importance algorithm based on distance evaluation (DF), and the information redundancy (IR) algorithm. To evaluate these different sensor placements we put forward the evaluation method. In addition, we can achieve the optimal sensor placement according to the index $R^{2}$.

### 3.1. The Workflow of Optimization Method

After obtaining the pressure data under different working conditions introduced in Section 2.2, it is first necessary to extract features from the pressure data to distinguish the flow-field parameters more effectively and avoid the dimension explosion. Secondly, although the extracted features can identify flow-field parameters from different perspectives, some features make an outstanding contribution. In contrast, other features have less relevant to some parameters. Therefore, it is necessary to calculate each feature’s importance and eliminate some features with a lower correlation with flow-field parameter identification. When optimizing sensor placement, feature importance is a significant parameter.

In this paper, two algorithms evaluate the importance of different features, MVF and DF. The evaluation result of the MVF algorithm on the normal distribution features is accurate, but the features’ distribution needs to be known in advance. Simultaneously, the DF algorithm is efficient in calculating the feature’s distance within different working conditions. Combining these two algorithms can obtain a more effective and more widely applicable algorithm.

The two algorithms, as mentioned above, only consider the ability of a single sensor to identify flow-field parameters. However, when multiple sensors collect data at the same time, redundant information may be generated. The existence of redundant information will reduce the efficiency of the learning algorithm. To accurately identify the flow-field parameters, we propose an IR algorithm to reduce the redundant information collected, combining the feature importance algorithm and the IR algorithm to obtain the optimal sensor placement.

**Figure 4:**
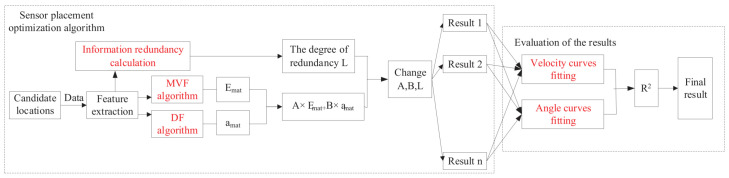
Sensor placement optimal algorithm.

The algorithm workflow to achieve the final sensor placement is shown in Algorithm 1. Figure 4 is the workflow of the optimization method.

**Algorithm 1** Sensor placement optimal algorithm.
**Step 1:** Calculate the feature importance using the MVF and DF algorithm. Supposing that the result are respectively $E_{mat}$ and $\alpha_{mat}$, weight factors are *A* and *B*.**Step 2:** Achieve a more efficient feature importance matrix *D*, which include the information of 31 sensor pairs’ feature importance.**Step 3:** Sum up the feature importance of each sensors.**Step 4:** Sort the sensor pairs according to the sum of feature importance from large to small.**Step 5:** Select the sensor pairs sensor the sorting results from front to back and satisfy the degree of information redundancy *L* as well.**Step 6:** Change the weight factors (A,B,L) of these three algorithms and achieve various sensor placements.**Step 7:** Calculate the evaluation indexes $R^{2}$ of fitted curves. The fitted curves include velocity and deflection angle fitting curves in the UF and KVS.**Step 8:** Choose the optimal sensor placement through the parameter $R^{2}$.


### 3.2. The Optimal Sensor Placement Algorithm

#### 3.2.1. Feature Extraction

For different flow-field speeds, the robotic fish’s deflection angle, the deviation distance of the robotic fish, the diameter of obstacles, etc., the hydrodynamics simulations simulation is performed. First, the features of the pressure data of different flow fields are extracted. Refer to the feature selection in the paper [23]; this paper has determined the same four frequency-domain features and ten time-domain features. Since all the features are extracted from the pair of sensors, the feature vector of the pair of sensors is constructed as (1).

(1) $$F=[T_{m,l},T_{m,r},f_{j,l},f_{j,r},f_{10}]$$

where $T_{m,l}$ and $T_{m,r}\left(m=1...4\right)$ respectively represent frequency-domain features extracted from the left and right sensors, $f_{j,l}$ and $f_{j,r}\left(j=1...9\right)$ respectively represent time-domain features extracted from the left and right sensors, $f_{10}$ is pressure difference between two sensors (left minus right).

#### 3.2.2. The Feature Importance Algorithm

1. The DF algorithm

Six pairs of experimental scenarios are conducted in Section 2.2 to simulate the natural underwater world. We change only one flow-field parameter in each team of experiments, such as speed, deflection angle, etc. Each experiment means one kind of working condition. The DF algorithm aims to calculate the distance evaluation factor, representing this feature’s ability to distinguish different working conditions.

Each experimental scenario in Figure 2 has conducted *C* times. The distance between experiments under the same working condition can be considered to be the distance within this working condition, and the distance between the three working conditions in a group, i.e., Figure 2(1–3), can be considered to be the distance between the working conditions. The number of working conditions is *M*, the number of features is *K*, the number of optional sensor pairs is *N*. In this paper, M=18,K=27,N=31. The evaluate algorithm method is as follows.

*Step 1:* Calculate the distance within the working condition.

(2) $$d_{i,j,k}\left(in\right)=\frac{1}{C(C-1)}\sum_{m,n=1}^{C}|F_{i,j,k}\left(m\right)-F_{i,j,k}\left(n\right)|$$

where m,n=1,2,...,C,m≠n, i=1,2,...,M, j=1,2,...,N, k=1,2,...,K, $F_{i,j,k}\left(m\right)$ and $F_{i,j,k}\left(n\right)$ represent the ith condition’s jth sensor’s mth and nth sample’s kth feature. Then the average distance within the *M* class working conditions is calculated.

(3) $$D_{j,k}\left(in\right)=\frac{1}{M}\sum_{i=1}^{M}d_{i,j,k}\left(in\right)$$

*Step 2:* Calculate the distance between the working conditions.

First calculate the average value of jth sensor’s kth feature.

(4) $$F_{j,k}\left(avr\right)=\frac{1}{M}\sum_{i=1}^{M}F_{i,j,k}$$

Then, the average value of the distance between the working conditions of the kth feature of the jth sensor is calculated.

(5) $$D_{j,k}\left(out\right)=\frac{1}{M}\sum_{i=1}^{M}|F_{i,j,k}-F_{j,k}\left(avr\right)|$$

where j=1,2,...,N, k=1,2,...,K, $F_{i,j,k}$ represent the average value of the kth feature of the jth sensor of the ith condition.

*Step 3:* Calculate the distance evaluation matrix.

Calculate the distance evaluate parameter of jth sensor’s kth feature.

(6) $$\alpha_{j,k}=D_{j,k}\left(out\right)/D_{j,k}\left(in\right)$$

The distance evaluate parameter $\alpha_{j,k}$ reflects the ability of the jth sensor’s kth feature to distinguish between *M* kinds of working condition. The greater the $\alpha_{j,k}$ is, the more sensitive the jth sensor’s kth feature to distinguish *M* kinds of working conditions.

*Step 4:* Remove the features with small distance evaluate parameter and data normalization.

To avoid the influence of small values on the experimental results, we can calculate the average value of distance evaluate parameter $\alpha_{avg}$.

(7) $$\begin{array}{c} \alpha_{avg}=\frac{1}{N\times K}\sum_{j=1}^{N}\sum_{k=1}^{K}\alpha_{j,k} \end{array}$$

Then remove the distance evaluate parameter smaller than $\alpha_{avg}$, which means $\alpha_{j,k}=0$. Normalize the distance evaluation matrix:

(8) $$\begin{array}{c} \alpha_{mat}\left(i,j\right)=\frac{\alpha_{mat}\left(i,j\right)-min\left(\alpha_{mat}\right)}{max\left(\alpha_{mat}\right)-min\left(\alpha_{mat}\right)} \end{array}$$

Then we can obtain the distance evaluate parameter matrix.

(9) $$\alpha_{mat}=\left[\begin{array}{ccc} \alpha_{1,1} & ... & \alpha_{1,K}\\ ... & ... & ...\\ \alpha_{N,1} & ... & \alpha_{N,K} \end{array}\right]$$

2. The MVF algorithm

In the two different working conditions, the feature’s average value is normalized by the features’ variance. If the average deviation value is considerable, this feature’s importance increases, which means it can distinguish two types of working conditions. If the average cannot be distinguishable, the importance of this feature diminishes. The following are the specific implementation steps of the method.

*Step 1:* The importance parameter *E* is calculated for each feature of each pair of sensors. $E_{mat}$ is the important matrix.

First, the importance of each group of sensors is calculated.

(10) $$\begin{array}{c} S_{i,j,k}=\sqrt{\frac{Var(F_{j,k})}{M}+\frac{Var(F_{i,k})}{M}} \end{array}$$

(11) $$\begin{array}{c} E_{i,j,k}\left(imp\right)=\frac{|mean\left(F_{j,k}\right)-mean\left(F_{i,k}\right)|}{S_{i,j,k}} \end{array}$$

where i,j=1,2,3...N,i≠j, k=1,2,3...K, $Var(F_{j,k})$ represents the variance of the kth feature of the jth sensor in *M* kinds of working conditions; average $\left(F_{j,k}\right)$ represents the average value of the kth feature of the jth sensor in *M* kinds of working conditions.

*Step 2:* Choose the maximum value of the kth feature of the ith sensor between the other (N−1) group of sensors as the feature importance parameter.

(12) $$\begin{array}{c} E_{j,k}\left(imp\right)=max\left(E_{i,j,k}\left(imp\right)\right) \end{array}$$

*Step 3:* To avoid the influence of small values on the experimental results, we can calculate the average value of feature importance $E_{avg}$.

(13) $$\begin{array}{c} E_{avg}=\frac{1}{N\times K}\sum_{j=1}^{N}\sum_{k=1}^{K}E_{imp} \end{array}$$

Then remove the distance evaluate parameter smaller than $E_{avg}$, which means $E_{avg}=0$. Additionally, we achieve a more efficient evaluate matrix $E_{mat}$.

(14) $$E_{mat}=\left[\begin{array}{ccc} E_{1,1} & ... & E_{1,K}\\ ... & ... & ...\\ E_{N,1} & ... & E_{N,K} \end{array}\right]$$

3. The synthesis of the two methods

The method of distance evaluation factor ends up with a N×K matrix $\alpha_{mat}$, and the method of characteristic importance end up with a N×K matrix $E_{mat}$, normalize the two matrices and add them. We can achieve a more efficient matrix *D*.

(15) $$\begin{array}{c} D=A\times E_{mat}+B\times \alpha_{mat} \end{array}$$

where A=1−B and A∈(0,1), *A* and *B* are the weights of these two algorithms.

#### 3.2.3. The IR Algorithm

When two features are highly dependent, if one of them is removed, the respective class-discriminative capabilities would not change much [25]. If two sensors collect similar features, the information efficiency between the two pairs of sensors may decrease. The different sensors’ redundant information should be as little as possible to ensure that the ALLS can achieve more efficient flow information. Inspired by the minimal redundancy used for feature selection in paper [25], the following IR algorithm is added to select mutually exclusive sensor pairs.

*Step 1:* Normalize the different features of different sensors.

*Step 2:* Calculate the probability of different sensors’ different features in different intervals.

After normalization, all data are between 0.1 and 1. Then, we divide them into nine intervals to calculate the occurrence probability of the Nth sensor’s Kth feature in different intervals.

*Step 3:* Calculate the information entropy $H_{1}$ for different features.

(16) $$\begin{array}{c} H_{1}\left(f_{i,j}\right)=-\sum P_{r}\left(f_{i,j}\right)\log P_{r}\left(f_{i,j}\right) \end{array}$$

where $P_{r}\left(f_{i,j}\right)$ represents the occurrence probability that the ith sensor’s jth features in the interval *r*.

*Step 4:* Calculate the conditional entropy between two different features of the sensors.

(17) $$P\left(f_{m,j,a}|f_{n,j,a}\right)=\frac{1}{\frac{F_{m,j,a}}{F_{n,j,a}}+\frac{F_{n,j,a}}{F_{m,j,a}}}$$

(18) $$\begin{array}{cc} \; & H_{2}\left(f_{m,j}|f_{n,j}\right)=\sum P\left(f_{n,j}\right)\sum_{a=1}^{M}P\left(f_{m,j,a}|f_{n,j,a}\right)\log P\left(f_{m,j,a}|f_{n,j,a}\right) \end{array}$$

where $F_{m,j,a}$, $F_{n,j,a}$ respectively represent the value of mth and nth sensor’s jth feature in the ath condition.

*Step 5:* Calculate the mutual information between the different features of two sensors.

(19) $$\begin{array}{c} L\left(f_{m,j}|f_{n,j}\right)=H_{1}\left(f_{m}\right)-H_{2}\left(f_{m,j}|f_{n,j}\right) \end{array}$$

*Step 6:* Add sensors’ different feature’s mutual information.

The result is the mutual information between two pairs of sensors. The more information the two sensors contain, the more redundant information is.

### 3.3. Evaluation of Different Sensor Placements

1. Change flow velocity in the UF and KVS, and fit the velocity curve.

Getting the flow velocity value from the original pressure signal is the critical issue in this paper [26] estimates the speed by the pressure difference between the fish’s tip and body. The pressure signal of the sensor at the tip of the fish is the total pressure, and the total pressure cannot directly obtain the flow velocity, as water is an incompressible flow in the Bernoulli equation:

(20) $$P=\rho gh+p_{0}+\frac{1}{2}\rho v^{2}$$

Since the water elevation does not change over time, and no changes in the bulk flow, so gravity is not considered, the formula (20) can be changed to:

(21) $$P=p_{0}+\frac{1}{2}\rho v^{2}$$

where *P* is the total pressure measured, $p_{0}$ is static pressure, ρ is fluid density, *v* is the fluid velocity.

(22) $$v=\sqrt{\frac{2(P-p_{0})}{\rho }}$$

In this formula, $p_{0}$ can be measured at the point on the fish’s head, where the flow speed is equal to the free flow speed. However, None of our sensors is mounted at this point, so $P-p_{0}$ cannot be directly measured. However, it can be replaced by $P-p_{a}$, where *P* is the mean value of pressure on both sides of the robotic fish, $p_{a}$ is the pressure at the nose of the robotic fish. However, it cannot be replaced directly. By trial, we find that there is a proportional relationship between the two data. Specifically, that is: $P-p_{0}=C_{s}\left(P-p_{a}\right)$. So, the velocity can be calculated as below equation.

(23) $$v=\sqrt{\frac{2C_{s}\left(P-p_{a}\right)}{\rho }}$$

2. Change the deflection angle in the UF and KVS, and fit the deflection angle curve.

In a uniform flow-field, if the robotic fish is deflected to a certain angle, the pressure value of the two sensors on both sides of the robotic fish will inevitably have a difference [13]. The pressure difference of the robotic fish increases as the deflection angle increases from $0^{\circ }$ to $90^{\circ }$. In our experiment, as the angle gradually increases, the pressure difference will increase sharply, which is similar to a quadratic function curve, not a linear relationship. Therefore, the corresponding relationship is set to second order to reduce the error in calculating the deflection angle.

(24) $$y=ax^{2}+bx+c$$

where *y* presents the pressure difference between a pair of sensors, *x* presents the deflection angle of the robotic fish.

Performance of the fitting curves is evaluated with $R^{2}$. $R^{2}$ is known as the goodness of fit, which is a parameter that evaluates the quality of the fitted model by comparing the estimated error with the sum of the squared errors. It is usually used to estimate the consistency of the fitted model.

## 4. Result Analysis

The data processing is coded in Matlab and implemented on a PC with a 3.10-GHz i7-5558U CPU, 4.00GB of RAM, and Windows 10 of 64 bits. The detailed result analysis is as follows.

### 4.1. Optimal Sensor Placement

#### 4.1.1. Change Three Parameters and Obtain Different Sensor Placements

Except for the sensor on the robot fish mouth, according to [23], setting up four pairs of sensors on the ALLS can obtain sufficient flow information and guarantee calculation efficiency. Therefore, optimal sensor placement is to select four sensor pairs from the optional 31 sensor pairs.

**Figure 5:**
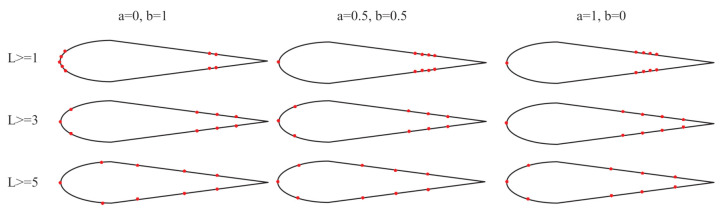
Change three parameters A,B,L, and obtain different sensor placements.

In Section 3.2.2, two algorithms are proposed to calculate the feature importance. The two algorithms are combined by changing the weight factors of the two feature importance algorithms. Moreover, the information redundancy degree is changed by changing the distance between the selected sensor pairs. By changing these three parameters, obtained when L≥1,2,3,4,5cm,A=0,0.1,0.2,0.3,…,1, we can obtain 55 sensor positions. Figure 5 shows a part of the sensor placements.

#### 4.1.2. Evaluation of Different Sensor Placements

To evaluate the optimal sensor placement algorithm, it is necessary to establish an evaluation model for different sensor placements. In Section 3.3, this paper introduces four fitting models: change velocity in the UF, change the deflection angle in the UF, change velocity in the KVS, and change the deflection angle in the KVS. Through the fitting models, we can evaluate the goodness of different sensor placements.

**Figure 6:**
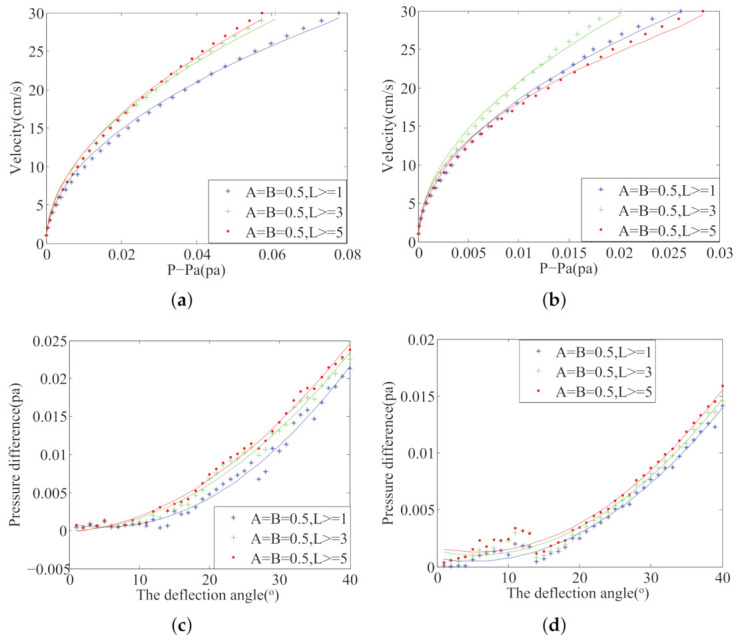
Model curve fitting with 4 conditions: (**a**) the UF Velocity condition, (**b**) the KVS Velocity condition, (**c**) the UF Angle condition, (**d**) the KVS Angle condition.

The ALLS collects and processes the pressure information in different flow fields, and it can identify the flow-field parameters. We conducted four experiments in Section 2.4. In each experiment, one flow parameter was changed, i.e., velocity, deflection angle. Figure 6 shows four kinds of fitting curves. Change three weight factors of the three algorithms mentioned above; multiple sensor placements are obtained. We selected three of them and drew four kinds of fitting curves. Assume that the selected sensor pairs are i,j,m, and *n*. In Figure 6a,b, the horizontal coordinate is $P-p_{a}$, where $P=(f_{1,i}+f_{1,j}+f_{1,m}+f_{1,n})/4$ and $p_{a}=f_{1,0}$. $f_{1,i}=\left(f_{1,i,l}+f_{1,I,r}\right)/2$, where $f_{1,i,l}$ and $f_{1,i,r}$ respectively represent the first time-domain feature extracted from the left and right sensors. $f_{1,0}$ represent the first time-domain feature extracted from the sensor that located at the snout of the robotic fish. In Figure 6c,d, the horizontal coordinate is the deflection angle between robotic fish’s body and flow. The vertical coordinates are the pressure difference between both sides of the fish’s body. $y=(f_{10,i}+f_{10,j}+f_{10,m}+f_{10,n})/4$.

**Figure 7:**
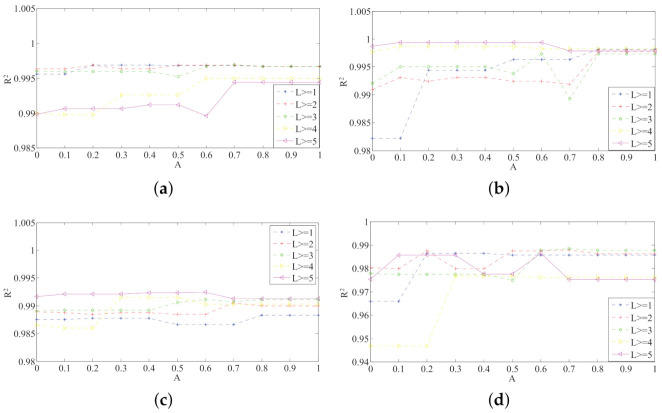
The parameter $R^{2}$ change with three weight parameters in 4 conditions: (**a**) the UF Velocity condition, (**b**) the KVS Velocity condition, (**c**) the UF Angle condition, (**d**) the KVS Angle condition.

We can achieve different kinds of sensor placements by changing three factors A, B, and L. Different sensor placements are evaluated by fitting four curves. We can calculate the evaluation parameter $R^{2}$ of the fitting curves. Figure 7 shows the parameter $R^{2}$ of different sensor placements. The x-coordinate of these four subgraphs is A, which is the weight of the MVF algorithm. B is the weight of the DF algorithm. L is the distance between the selected sensor pairs. The dotted lines in the graphs are the $R^{2}$ value of different sensor placements selected by the optimal sensor placement algorithms. These four subgraphs show the goodness of different sensor placements. The larger $R^{2}$ indicates that this sensor placement can predict flow parameters more accurately.

#### 4.1.3. The Optimal Sensor Placement

**Figure 8:**
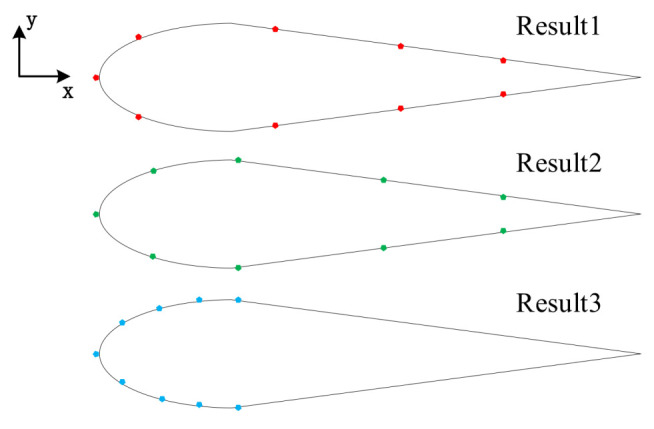
Sensor placement: the optimal placement; the uniform placement; the uniform placement in the head.

Using the curve fitting method, we calculate the evaluation parameter $R^{2}$, and the optimal sensor placement is obtained according to $R^{2}$. Add up the evaluation parameter $R^{2}$ of the above four fitting models, choose the sensor placement with the largest $R^{2}$ as the optimal sensor placement. After calculation, we obtain Result 1 subject to the condition that A =0.3, B =0.7, and L ≥5. In the recent study, sensors in the ALLS are often distributed evenly, which means pairs of sensors are placed at the halfway point of the robotic fish’s head, and another two pairs of sensors are placed at the one-third point and two-thirds point of the robotic fish’s tail. This sensor placement is Result 2. In paper [4], two pairs of sensors are distributed evenly on the robotic fish’s head. To imitate this kind of sensor placement, we place four pairs of sensors evenly on the head of the robotic fish, and this sensor placement is Result 3. Figure 8 shows three kinds of sensor placements. Sensors’ coordinates of these three sensor placements are shown in Table 3.

### 4.2. Performance Validation

**Figure 9:**
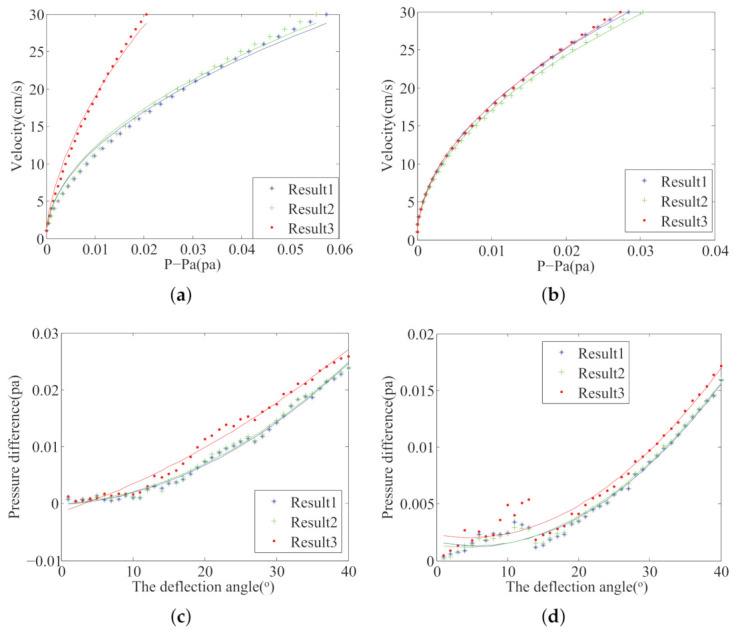
Experiment curves fitting in 4 conditions: (**a**) the UF Velocity condition, (**b**) the KVS Velocity condition, (**c**) the UF Angle condition, (**d**) the KVS Angle condition.

**Figure 10:**
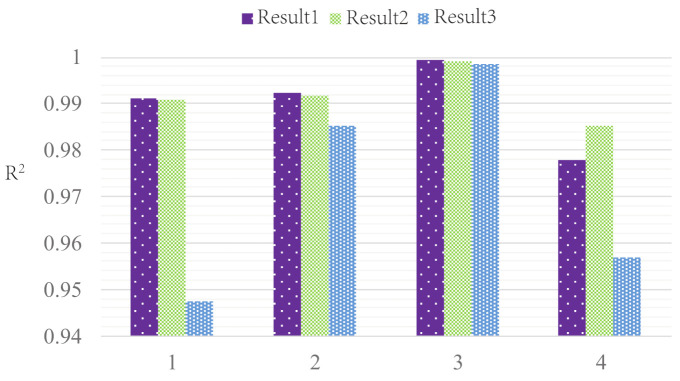
Three results’ curves fitting parameter $R^{2}$ in four conditions.

To verify the effectiveness of the optimal sensor placement algorithm, we conducted four groups of experiments. The experimental condition is shown in Section 2.4. In each experiment group, we change the flow velocity or the deflection angle in the UF or KVS. The features analyzed in Figure 9 are the same as Figure 6. The curve fitting method is used to predict water flow parameters; the evaluation parameter $R^{2}$ of the fitting curve was calculated to evaluate the accuracy of different sensor placements’ parameter prediction ability. These three sensor placements are evaluated by comparing the curve fitting indexes $R^{2}$. Figure 9 shows four fitting curves of three sensor placements. Figure 10 shows the evaluation parameter $R^{2}$ of three sensor placements in four conditions. 1–4 in Figure 10 respectively represent UF Velocity condition, KVS Velocity condition, UF Angle condition, and KVS Angle condition. Table 4 shows the numerical comparison results of each case in Figure 10. As can be seen, due to the disturbance in the experiment, the fitting curves are not good enough compared with the simulation result. However, in general, Results 1 is superior to Result 2 and Result 3. Only in the KVS Angle condition, the evaluation parameter $R^{2}$ of Result 1 is smaller than Result 2. When the deflection angle is less than $15^{\circ }$, the curve fitting result is not good enough in the KVS Angle condition. We think that the fitting model of changing the angle in KVS is not accurate enough to predict the deflection angle because of the vortex’s disturbance.

### 4.3. Discussion

**Figure 11:**
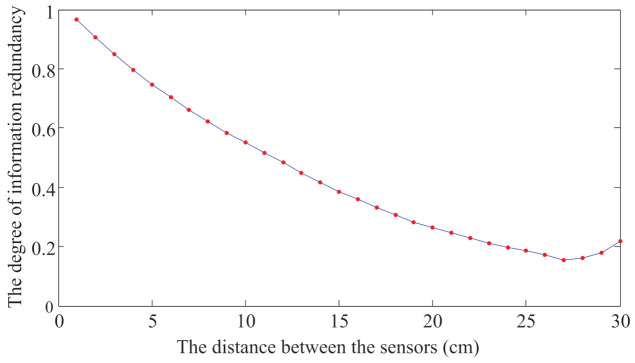
The relationship between information redundancy and the distance between sensors.

According to the algorithm in Section 3.2.3, the result of the information redundancy between different sensor pairs is calculated and shown in Figure 11. The horizontal coordinate represents the distance between two sensor pairs, and the vertical coordinate represents the degree of information redundancy between two sensor pairs. As the figure shows, the further the distance between two sensor pairs are, the fewer redundant information is. The information redundancy between two sensor pairs can be divided into five standards, and the corresponding distances between sensor pairs are shown in Table 5.

## 5. Conclusions

To control robotic fish efficiently, the ALLS must accurately distinguish flow-field type, flow velocity, deflection angle, and other flow parameters. The sensor placement of the ALLS has a significant influence on flow parametric recognition. An inappropriate sensor placement may result in information loss or redundancy, leading to mistakes in flow parametric recognition. Therefore, this paper presents a sensor placement optimal algorithm for the ALLS.

This paper puts forward a sensor placement optimal method based on the feature importance and information redundancy. The DF algorithm and the MVF algorithm can calculate the importance of features, respectively. Both are efficient. Through the feature importance algorithm, we can choose sensors with better working condition identification capabilities. Moreover, the IR algorithm is proposed to evaluate the degree of information redundancy between sensors. Using this algorithm, multiple sensor information can be combined to identify the working conditions. We can achieve the optimal sensor placement through these three algorithms, the DF, the MVF, and the IR. Simulation and experiments show that the sensor placement chosen by this method can provide higher accuracy for flow-field parametric identification. Compared with the uniform sensor placement used in the recent studies, we find that the optimal sensor placement can provide higher accuracy to identify the flow parameters, i.e., velocity and deflection angle. The optimal sensor placement of the ALLS improves the accuracy of flow-field parametric identification, which helps to realize the closed-loop control of the robotic fish. To some extent, the resemblance to the shape of some aquatic robots (robotic boats or robotic fish), so this optimal method will be useful for the ALLS in other robotic fish.

In the future, we will continue this study by considering the size and shape of the robotic fish and more investigated scenarios to verify the effectiveness of the layout optimization method. Moreover, we will study sensor placement optimization in three-dimensional space to identify the three-dimensional flow-field’s information.

## Figures and Tables

**Table 1:** Simulation experiment conditions and hydrodynamic parameters.

Experiment Conditions			
Uniform flow			
Flow Speed (cms${}^{-1}$)	10	20	30
Deflection angle ${(}^{\circ })$	0	15	30
Karman vortex street			
Flow Speed (cms${}^{-1}$)	10	50	200
Deflection angle ${(}^{\circ })$	0	15	30
Distances deviated from center (mm)	0	5	10
Cylinder diameter (mm)	25	50	75
Hydrodynamic parameters			
Strouhal number	0.21		
Spacing ratio	0.41±0.08		

**Table 2:** Experiment conditions and flow parameters.

	Experiment 1	Experiment 2	Experiment 3	Experiment 4
Flow speed (cm/s)	1–30	100	1–30	100
Deflection angle (${}^{\circ }$)	0	0–40	0	0–40
Distance deviated from center (mm)	/	/	0	0
Cylinder diameter (cm)	/	/	10	10

**Table 3:** Coordinates of sensors’ positions.

	1st Sensor	2nd Pair	3rd Pair	4th Pair	5th Pair
Result 1	(400,0)	(442.1,±26.3)	(525.6,±39.6)	(574.0,±28.6)	(622.5,±17.6)
Result 2	(400,0)	(450.6,±31.6)	(506.2,±44.0)	(564.4,±30.8)	(622.5,±17.6)
Result 3	(400,0)	(425.3,±15.8)	(450.6,±31.6)	(476.8,±45.4)	(506.2,±44.0)

**Table 4:** The fitting parameter $R^{2}$ in four conditions.

R${}^{2}$	Condition 1	Condition 2	Condition 3	Condition 4
Result 1	0.9912	0.9923	0.9996	0.9780
Result 2	0.9907	0.9919	0.9992	0.9857
Result 3	0.9476	0.9853	0.9984	0.9569

**Table 5:** The calculated information redundancy.

The Distance between the Sensors (cm)	L
1	96.48%
2	90.65%
3	84.77%
4	79.55%
5	74.55%
